# Evaluation of the practical application of the category-imbalanced myeloid cell classification model

**DOI:** 10.1371/journal.pone.0313277

**Published:** 2025-01-30

**Authors:** Zhigang Hu, Aoru Ge, Xinzheng Wang, Cuisi Ou, Shen Wang, Junwen Wang

**Affiliations:** 1 School of Medical Technology and Engineering, Henan University of Science and Technology, Luoyang, China; 2 School of Mechanical and Electrical Engineering, Henan University of Science and Technology, Luoyang, China; Stanford University, UNITED STATES OF AMERICA

## Abstract

The incidence of acute myeloid leukemia (AML) is increasing annually, and timely diagnostic and treatments can substantially improve patient survival rates. AML typing traditionally relies on manual microscopy for classifying and counting myeloid cells, which is time-consuming, laborious, and subjective. Therefore, developing a reliable automated model for myeloid cell classification is imperative. This study evaluated the performance of five widely-used classification models on the largest publicly available bone marrow cell dataset (BM). However, the accuracy of the classification model is significantly affected by the imbalance in the distribution of bone marrow cell types. To address this issue, this study analyzed five different Loss functions and seven different attention mechanisms. When the classification models is chosen, Swin Transformer V2 was found to perform the best. However, the lightweight model RegNetX-3.2gf had significantly fewer parameters and a significantly faster inference speed than Swin Transformer V2, and its F1 Score was only 0.032 lower than that of Swin Transformer V2. Accordingly, RegNetX-3.2gf is strongly recommended for practical applications. During the evaluation of Loss function and attention mechanism, the Cost-Sensitive Loss Function (CS) and the channel attention mechanism Squeeze-and-Excitation Networks (SE) demonstrated superior performance. The optimal model (RegNetX-3.2gf + CS + SE) achieved an average precision of 68.183%, an average recall of 63.722%, and an average F1 Score of 65.155%. This model exhibited significantly improved performance compared to the original dataset results, achieving an enhancement of 17.183% in precision and 10.655% in the F1 Score. Finally, the class activation maps demonstrate that our model focused on the cells themselves, especially on the nucleus when making classifications. It proved that our model was reliable. This study provided an important reference for the study of bone marrow cell classification and a practical application of the model, promoting the development of the intelligent classification of AML.

## Introduction

Acute myeloid leukemia (AML) is a highly heterogeneous hematologic malignancy marked by the proliferation of immature leukemic cells in the blood and bone marrow, consequently reducing the available space for healthy hematopoietic cells and leading to bone marrow failure. Among the various types of leukemia, AML exhibits the highest incidence [1]. Data from the SEER database [2] of the National Cancer Institute indicate an annual incidence rate of 4.2 and a mortality rate of 2.7 per 100,000 individuals. Furthermore, the 5-year relative survival rate of AML patients has been reported to be 31.9%. Timely and accurate diagnosis is essential for implementing effective treatment strategies, which in turn enhances patient survival rates.

The diagnostic methodology endorsed by the World Health Organization for AML relies on morphological analysis [3], which involves the examination of peripheral blood and bone marrow smears. These smears are stained and then examined under a light microscope to scrutinize cellular structures, including the nucleus, cytoplasm, and nucleolus, to identify the cell type. However, this process is cumbersome, time-consuming, and easily influenced by the experiences and subjective factors of the experts on morphological features, making standardization challenging. With the development of information technology, many researchers apply artificial intelligence to the medical field to obtain some achievements, including the diagnosis of skin diseases or cervical cancer, since deep learning shows great potential in medical diagnosis and treatment. Therefore, developing a potential machine learning for cell classification significantly accelerates the diagnostic process and ensures the reproducibility of results, facilitating verification and review.

Progress in blood cell classification in peripheral blood smears has advanced significantly with the development of instruments such as DM9600, DI-60, Cobas M511, and Vision Hema [4]. However, research on bone marrow smears remains in its early stages. Acquiring bone marrow smear data is difficult, laborious, costly, and time-consuming. Publicly accessible datasets are scarce. The models trained on self-constructed datasets often suffer from poor generalization due to variables such as staining and lighting conditions. Bone marrow smears contain numerous cells at various stages of differentiation or maturation, with often unclear boundaries, making classification challenging. For example, Promonocytes, which are clinically counted as blasts, are prone to human error and are typically excluded from classification [5]. As leukemia classifications become more specific, merging cell types is no longer adequate to meet diagnostic needs. Accurate classification of monocyte subgroups (monocytes, promonocytes, and blasts) is essential for diagnosing and classifying monocytic leukemias [6]. Additionally, the distribution of bone marrow cells is highly heterogeneous due to varying disease prevalence and cell function, leading to an imbalance among cell populations. For instance, lymphocytes are abundant in bone marrow smears, whereas eosinophils are scarce, exhibiting a long-tail distribution. Models tend to be more sensitive to categories with more samples when classifying cell types, often overlooking categories with fewer samples, which can result in overfitting and poor performance on rare cell types. However, these ignored cell types may play a crucial role in disease diagnosis and classification. Thus, selecting appropriate classification models and developing effective training strategies are essential to address the challenges posed by long-tail distribution in bone marrow datasets.

At present, there are three types of solutions to the long-tail class imbalance [7]: class rebalance, information enhancement, and module improvement. Among them, loss adjustment of category rebalance, migration learning of information enhancement, and module improvement are the three most commonly used methods. Based on this, this study aimed to investigate the effects of different Loss and attention mechanisms on the classification performance of bone marrow cells. The main contributions of this study are as follows:

1. Evaluation of the performance of five widely-used classification models on a publicly available bone marrow smear cell classification dataset (BM)
2. Assessment of the effects of various loss functions on categorizing bone marrow cells with category imbalance.
3. Evaluation of the effects of various attention mechanisms on classifying bone marrow cells with category imbalance.
4. Determination of whether the model was reliable when making classification decisions.

## Related work

### Cell classification

The systematic analysis of bone marrow cell classification began with the application of machine learning techniques, such as support vector machines, random forests, and hierarchical decision trees. However, traditional machine learning methods rely on manually engineered features, which results in a significant workload. In contrast, deep learning can autonomously extract complex features from data. By creating a multi-layered neural network for complex data analysis, deep learning reduces the manual workload. Consequently, researchers increasingly prefer using it in the classification of bone marrow cells [8].

Currently, the predominant source of data for the classification of bone marrow cells is self-built datasets. Jin et al. [9] developed an automated system for the digital scanning of bone marrow smears, creating a 27-layer classification network capable of identifying 12 common types of bone marrow cells. The dataset was based on bone marrow smears from Sir Run Run Shaw Hospital, affiliated with Zhejiang University School of Medicine. The system achieved an overall accuracy of 90.1% (95% CI, 89.8–90.5%) in automated cell categorization. Guo et al. [10] curated a dataset with 7,484 bone marrow cells and introduced a category-balanced (CB) categorization method to mitigate category imbalance. The method achieved an average accuracy of 84.53%, sensitivity of 84.44%, and specificity of 99.29% in classifying 15 distinct types of bone marrow cells. They also analyzed the outcomes by using the Guided Grad-CAM visualization technique and a confusion matrix. Wang et al. [11] developed an efficient and fully automated hierarchical deep learning framework designed to identify 16 distinct cell types, including megakaryocytes, mitotic cells, and various stages of erythropoietic cells. The framework was evaluated on a dataset with 12,426 annotated units, achieving a recall of 0.905 ± 0.078 and an accuracy of 0.989 ± 0.006. Validation on an independent dataset yielded the final recall and accuracy scores of 0.842 and 0.988, respectively.

Existing public datasets serve as the second major data source. Choi et al. [12] developed an automatic white blood cell classification and counting system for bone marrow smear images by using a two-stage convolutional neural network (CNN). To train and test this system, a dataset of 2,174 bone marrow images was used. Their two-stage CNN could classify the images into 10 distinct myeloid and erythroid series, achieving 97.06% accuracy, 97.13% precision, 97.06% recall, and an F1 Score of 97.1%. Furthermore, Matek et al. [13] established a bone marrow cell classification dataset (BM) [14] with 171,374 annotated images from 945 patients with various blood disorders. This dataset is currently the largest publicly available dataset in terms of patient numbers, diagnostic cases, and variety of included cells. The authors employed ResNeXt-50 to classify 21 bone marrow cell types, with data augmentation and cross-validation addressing category imbalance. Both stringent and lenient evaluation strategies were employed to handle the difficulty of distinguishing neighboring categories. Stringent evaluation resulted in a maximum single-category classification precision of 92% and a recall rate of 91%. Under lenient conditions, precision rose to 95%, with recall remaining at 91%. The classification decisions of ResNeXt-50 were further analyzed using SmoothGrad and Grad-CAM to identify regions of interest during training. Additionally, Alshahrani et al. [15] selected seven categories from the BM dataset–ABE, BAS, FGC, HAC, LYI, KSC, and OTH–and expanded it to 7,065 samples via data enhancement. Among five transfer learning models, DenseNet121 showed the best performance. Model optimization, including tuning the optimizer, adjusting batch size, and adding attention mechanisms, improved the results. The optimized DenseNet121 achieved 97.01% classification accuracy. Glüge et al. [16] evaluated four state-of-the-art CNN architectures on the BM dataset, analyzing both in-domain and out-of-domain datasets to assess the impact of pre-trained models. The pre-trained RegNet_Y_32gf achieved average accuracy, recall, and F1 Score of 0.787 ± 0.060, 0.755 ± 0.061, and 0.762 ± 0.050, respectively. Compared to training ResNeXt-50 from scratch, this model achieved a 53.5% increase in accuracy and a 7.3% improvement in recall. Activation maps were also employed to explain the predictions of the model.

Peng et al. [17] combined self-constructed and public datasets to evaluate the generalization of their model. By incorporating a novel dual attention gate (DAG) within DenseNet, they developed DAGDNet, which aims to enhance precision and recall in neural network based cell classifiers. The model was trained and tested on both the self-constructed CMU dataset and the public BM dataset, achieving precision rates of 90.3% and 88.1%, respectively.

Regardless of whether the dataset is self-constructed or publicly available, models perform better when the cells have large sample sizes and clear features. However, models often struggle to identify scarce cell types, such as lymphoblasts and faggot cells, leading to lower classification accuracy. Therefore, addressing the long-tail distribution of the dataset can improve the ability of the model to classify minority classes, thereby enhancing overall performance.

### Imbalance in the long-tail category

**Fig 1:**
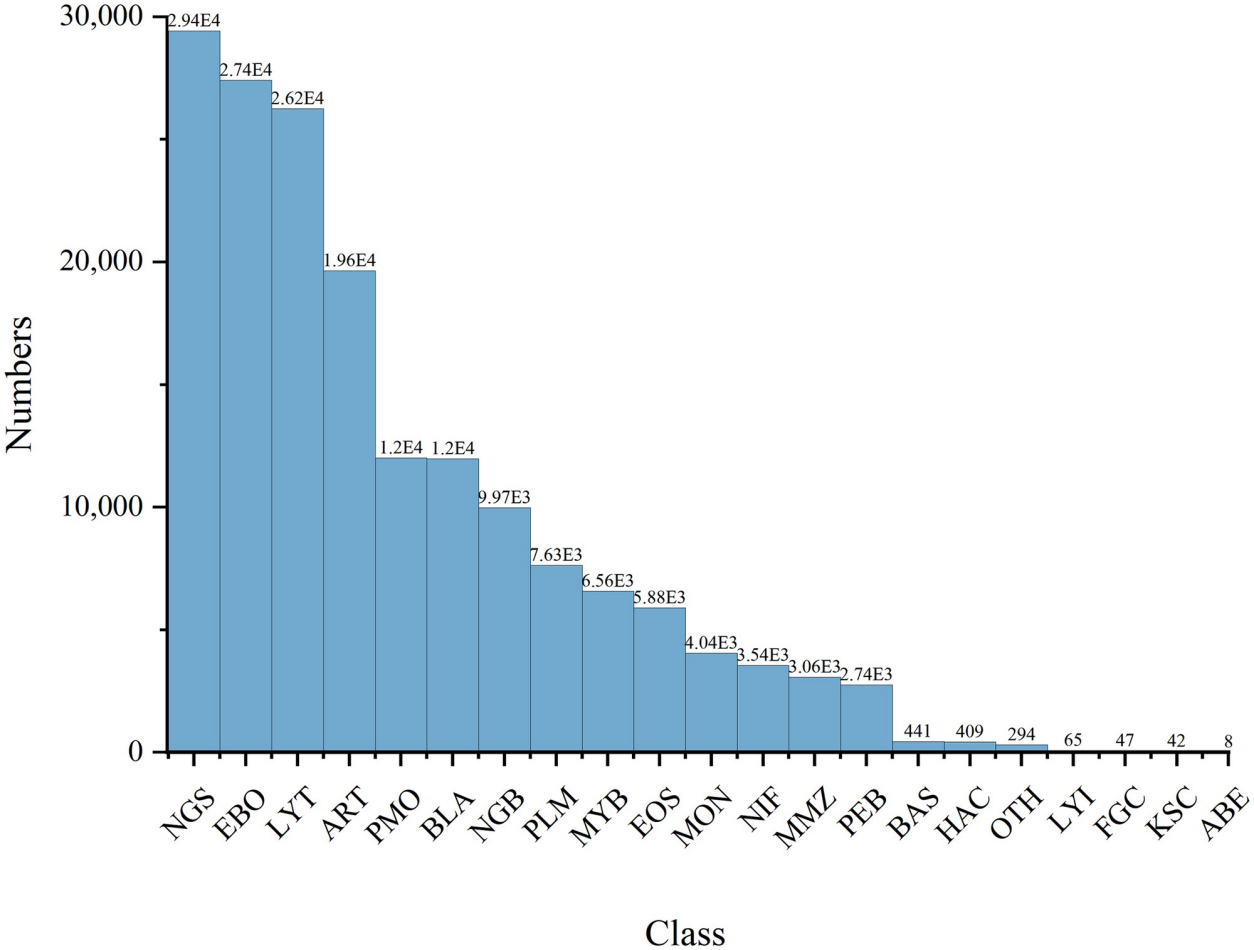
Distribution of the dataset.

Long-tailed category imbalance refers to a distribution where a few categories have many samples, while most have few. This pattern is common in real-world scenarios, such as book sales and video views. Bone marrow cells also exhibit a typical long-tailed imbalance (Fig 1). Training a classification model on a long-tail dataset often leads to overfitting on the head categories and underfitting on the tail categories. To address this issue, many researchers have conducted extensive studies in this area in recent years.

The most common method to solve this problem is to balance the number of training samples of different classes in the training process. Ren et al. [18] proposed a Meta Sampler that employs meta-learning to determine optimal sampling rates while using Balanced Softmax to avoid overbalancing. This approach achieves state-of-the-art performance on long-tailed image classification datasets. Park et al. [19] introduced a novel IB loss function during balanced training to mitigate the impact of category imbalances on classification, reducing the influence of categories with large sample sizes and thereby enhancing the efficacy of the classifier.

Furthermore, model performance can be improved by incorporating supplementary information into the training process. Wang et al. [20] used transfer learning to transfer knowledge from the dominant class to the tail class, enhancing model performance in the target domain. Chu et al. [21] suggested data mixing to tackle challenges in long-tailed learning and introduced a rebalanced mixing strategy specifically designed to improve tail class performance.

Improving the network module can also enhance classification performance on long-tail distribution datasets. Wu et al. [22] proposed a Deep Real-Time Classifier that categorizes each sample according to the level of competence via random tree sampling. It simulates classification conditions and uses a rejection mechanism to discard samples at different classification levels, retaining more information across all classes. Additionally, Zhou et al. [23] introduced the Unified Bilateral Branch Network, which handles both representation and classifier learning simultaneously. The network comprises two branches: one utilizes uniform sampling to replicate the original long-tail training distribution, and the other employs a reverse sampler to increase the representation of tail class samples, thereby improving their model’s performance. During training, predictions from both branches are dynamically integrated to improve the learning efficacy of the classifier.

## Methodology

### Dataset introduction and processing

This study used the publicly accessible BM dataset provided by the Munich Leukemia Laboratory. In terms of patient count, cell type diversity, and the range of cell types included, it is the largest dataset of its kind currently available. Consequently, the findings derived from training with this dataset hold significant practical relevance and reference value. The dataset encompasses 171,374 expertly annotated bone marrow cells derived from the smears of 945 patients. The data, acquired by Munich Leukemia Laboratory, were generated using a bright-field microscope with 40 × magnification and oil immersion. All the samples were processed at Munich Leukemia Laboratory, and the images were standardized to 250 × 250 pixels. The dataset comprises 21 categories, with each category and its corresponding count detailed in Table 1. Notably, segmented neutrophils constitute the largest type, totaling 29,424 samples, whereas abnormal eosinophils represent the smallest type, with only 8 samples. This highlights a high imbalance of samples.

**Table 1:** Description of the dataset.

Abbreviation	Meaning	Numbers
**ABE**	Abnormal eosinophil	8
**ART**	Artefact	19,630
**BAS**	Basophil	441
**BLA**	Blast	11,973
**EBO**	Erythroblast	27,395
**EOS**	Eosinophil	5,883
**FGC**	Faggot cell	47
**HAC**	Hairy cell	409
**KSC**	Smudge cell	42
**LYI**	Immature lymphocyte	65
**LYT**	Lymphocyte	26,242
**MMZ**	Metamyelocyte	3,055
**MON**	Monocyte	4,040
**MYB**	Myelocyte	6,557
**NGB**	Band neutrophil	9,968
**NGS**	Segmented neutrophil	29,424
**NIF**	Not identifiable	3,538
**OTH**	Other cell	294
**PEB**	Proerythroblast	2,740
**PLM**	Plasma cell	7,629
**PMO**	Promyelocyte	11,994
**Add up the total**	171,374	

The dataset was split into training and test sets in an 80:20 ratio, with 20% of the training data used for validation. The primary objective was to evaluate the classification performance of the model on the original dataset via minimal preprocessing. The augmentation techniques employed for classifying bone marrow cells included random cropping, resizing the images to 224 × 224 pixels, horizontal flipping of the images with a probability of 0.5, and adjustments to image illumination.

### Introduction to the model

This study aimed to advance the development of the bone marrow cell classification task model to provide a more extensive and representative result reference. Therefore, a comparative analysis of multiple types of models is needed to assess their strengths and weaknesses in the bone marrow cell classification task, thus providing a more valuable reference for model selection.

Below are introductions to the five classic classification models compared in this study, with the model parameters detailed in Table 2. DenseNet [24] features a densely connected architecture, where each layer is directly linked to all preceding layers. This structure promotes efficient information flow, reduces the vanishing gradient problem, and maintains a relatively low parameter count. ResNext [25], a variation of ResNet [26], employs a highly modular design consisting of multiple residual blocks with uniform topologies. The concept of cardinality defines the number of replicated subnetwork units within each residual block, enhancing computational efficiency while mitigating excessive network depth through the introduction of branches. MobileNetV3 [27] is a lightweight model characterized by fewer parameters, reduced computational load, and faster inference times than the other models, making it particularly suitable for applications with limited storage capacity and power constraints, such as mobile and embedded systems. RegNet [28] has an innovative design that optimizes performance efficiency across a range of tasks by modulating the complexity of the network architecture. Swin Transformer V2 [29] incorporates scaled cosine attention and adjusts the position of the layer normalization based on V1 [30], facilitating adaptation to various image resolutions and sizes. It displays robust performance across multiple visual tasks. These models exhibit strong performance across numerous benchmark datasets, showcasing commendable classification and generalization capabilities.

**Table 2:** Models used in this study and the number of training parameters.

Models	Params
Densenet-201	20,013,928
ResNext-50	25,028,952
Mobilenetv3	**2,542,856**
RegNetX-3.2gf	15,296,627
Swin Transformer V2	90,216,536

**Fig 2:**
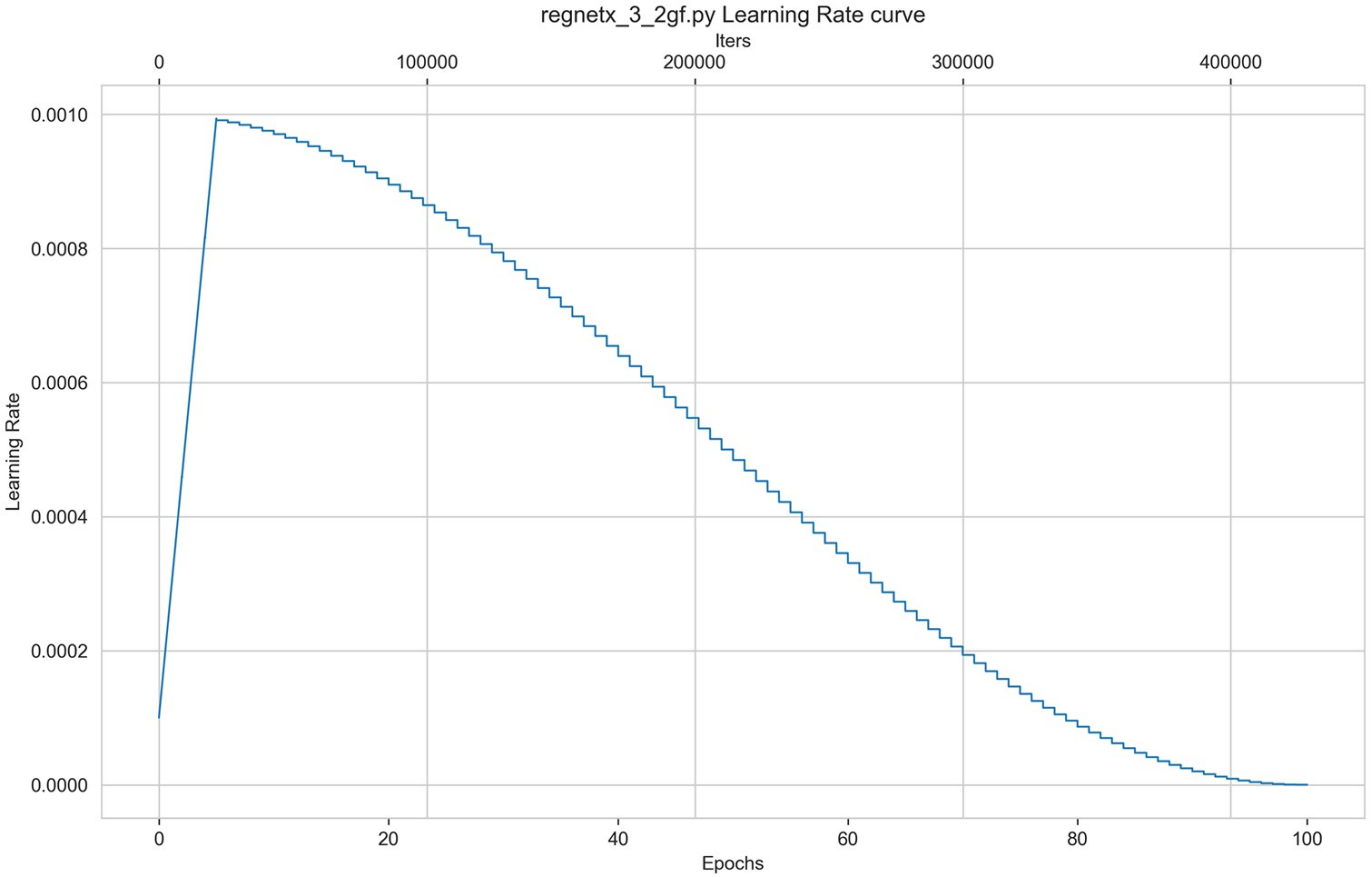
Learning rate decline strategy.

All the models were trained for 100 epochs with a batch size of 32. The implementation utilized PyTorch’s stochastic gradient descent (SGD) optimization, maintaining a fixed momentum of 0.9. The initial learning rate was set to 0.001, and the learning rate-decay strategy is depicted in Fig 2.

### Loss function

The loss function quantifies the difference between the predicted outcomes and the actual outcomes. Selecting an appropriate loss function is a common strategy for addressing the challenge of long-tailed distributions, as it helps rebalance categories by adjusting the loss values assigned to each category during training, thereby mitigating the effects of dataset imbalance. In this study, we evaluated five commonly used loss functions specifically designed for imbalanced datasets.

Suppose the input example is *x*, with a corresponding label *y* ∈{1, 2, …, *C*}, where *C* represents the number of distinct classes. The output produced by the fully connected layer of the model is *z* = [*z*_1_, *z*_2_,…, *z*_*c*_]^*T*^, defined as $z_{i}^{t}$ where

$$z_{i}^{t}=\left\{\begin{array}{cc} z_{i}, & if\;i=y.\\ -z_{i}, & otherwise \end{array}\right.$$ (1)

Here, *i* ∈ {1, 2, …, *C*}. Let $p_{i}^{t}$ denote the probability of being categorized as *i*; this probability is given by $p_{i}^{t}=sigmoid\left(z_{i}^{t}\right)=1/[1+\text{exp}\;\left(-z_{i}^{t}\right)]$.

The cross-entropy loss function (CE) is widely utilized in classifiers. This study adopted CE as the baseline for evaluating subsequent loss functions. Its fundamental principle involves minimizing the loss value by computing the cross-entropy between the probability distribution predicted by the model and the true labels. This process gradually aligns the probability distribution of the model with the actual probability distribution, as shown in Eq 2.


$$CE\left(z,y\right)=-\sum_{i=1}^{C}\text{log}\;\left(p_{i}^{t}\right)$$ (2)

However, in cases of imbalanced data, utilizing CE often causes the neural network to learn toward the largest disparity between the correct and incorrect labels during training, thus increasing the risk of overfitting. To mitigate this issue, Szegedy et al [31] proposed a regularization method designed to approximate the boundary effects of label loss during training, incorporating this approximation as a regularization term within the loss function to reduce model overfitting. This regularization technique is known as label smoothing loss (LSL), as represented in Eq 3.

$$LSL\left(z,y\right)=-\left(\left(1-\varepsilon \right)\delta_{c,y}+\frac{\varepsilon }{C}\right)\sum_{i=1}^{C}\text{log}\;\left(p_{i}^{t}\right)$$ (3)

Where *δ*_*c*,*y*_ represents the Dirac delta function, it equals 1 only if *c* = *y*; otherwise, it is 0. This function helps reduce sensitivity to uncertainties or noise during the training phase, thereby mitigating overfitting. The parameter *ε* represents the degree of label smoothing. By employing $\frac{\varepsilon }{C}$, label smoothing can be uniformly distributed across all categories. The label smoothing loss (LSL) is that incorporates an additional smoothing term into CE, and this strategy works well for datasets with noise or uncertain labels.

Furthermore, to address data imbalance, Lin et al. [32] introduced the Focal Loss (FL) concept. This approach incorporates an adjustable modulation factor into the cross-entropy loss term ${\left(1-p_{i}^{t}\right)}^{\gamma }$, where the focusing parameterγis a tunable value satisfying γ≥ 0. FL is calculated as in Eq 4.


$$FL\left(z,y\right)=-\alpha_{t}\overset{C}{\underset{i=1}{\sum }}{\left(1-p_{i}^{t}\right)}^{\gamma }\;\text{log}\left(p_{i}^{t}\right)$$ (4)

In practical applications, it is common to employ the *α* variation of FL, where *α*_*t*_ represents the weighting coefficient. When a class is misclassified and $p_{i}^{t}$ is lower, the modulation coefficient approaches 1, and the loss remains unaffected. Conversely, when $p_{i}^{t}$ approaches 1, the coefficient tends toward 0, reducing the impact of loss for correctly classified classes. By introducing this penalty factor, the model can reduce its focus on simple categories and concentrate more on difficult ones, thereby mitigating the adverse effects of data imbalance.

Cui et al. [33] argued that the application of a category rebalancing strategy to mitigate challenges associated with long-tailed distribution often diminishes the incremental benefits of newly added datasets as sample size increases. They introduced the concept of the "effective sample," calculated using the equation $\frac{1-\beta^{n_{y}}}{1-\beta },$ where *n*_*y*_ represents the effective number of samples for category *y*, and *β* ∈ [0, 1) is a hyperparameter. This approach facilitates loss rebalancing by incorporating effective sample counts for each category, thereby impacting the category-specific loss. The category balancing loss (CB), based on FL, is calculated using Eq 5.


$$CB_{focal}\left(z,y\right)=-\frac{1-\beta }{1-\beta^{n_{y}}}\overset{C}{\underset{i=1}{\sum }}1-{p_{i}^{t}}^{\gamma }\;\text{log}\left(p_{i}^{t}\right)$$ (5)

In determining effective samples, the weight factor $\alpha_{t}=\frac{1-\beta }{1-\beta^{n_{y}}}$ in the loss function can be adjusted by modifying parameters *β* and *γ*. Such adjustments can mitigate class imbalance by allowing the model to adjust the focus on both difficult and easy samples.

Galdran et al. [34] introduced the concept of "Cost-Sensitive Loss (CS)," which incorporates an additional term into the standard loss function to impose a greater penalty when there is a significant discrepancy between the predicted and actual outcomes. In this study, CE is used as the standard loss function (Eq 6).

$${CS}_{ce}\left(z,y\right)=-\alpha_{t}\sum_{i=1}^{C}\;\text{log}\;\left(p_{i}^{t}\right)+\lambda \left\langle z,M(y,∙\left.)\right\rangle \right.$$ (6)

Where $M=(M^{\left(2\right)}+I-M_{opht}^{*})/2$. Matrix *M* represents the cost-sensitive matrix employed to evaluate the interrelationships among various categories. It is structured as a (*C*, *C*) matrix, where *C* represents the number of categories. Symbol *I* represents the identity matrix, and $M_{opht}^{*}$ represents the probabilistic cost-sensitive matrix, indicating the probability distribution of each category within the cost-sensitive matrix. By integrating the probabilistic cost-sensitive matrix with the loss function, the model can prioritize classification accuracy for significant categories. This approach enhances the performance of the model, particularly in cases of category imbalance.

### Attention mechanisms

**Fig 3:**
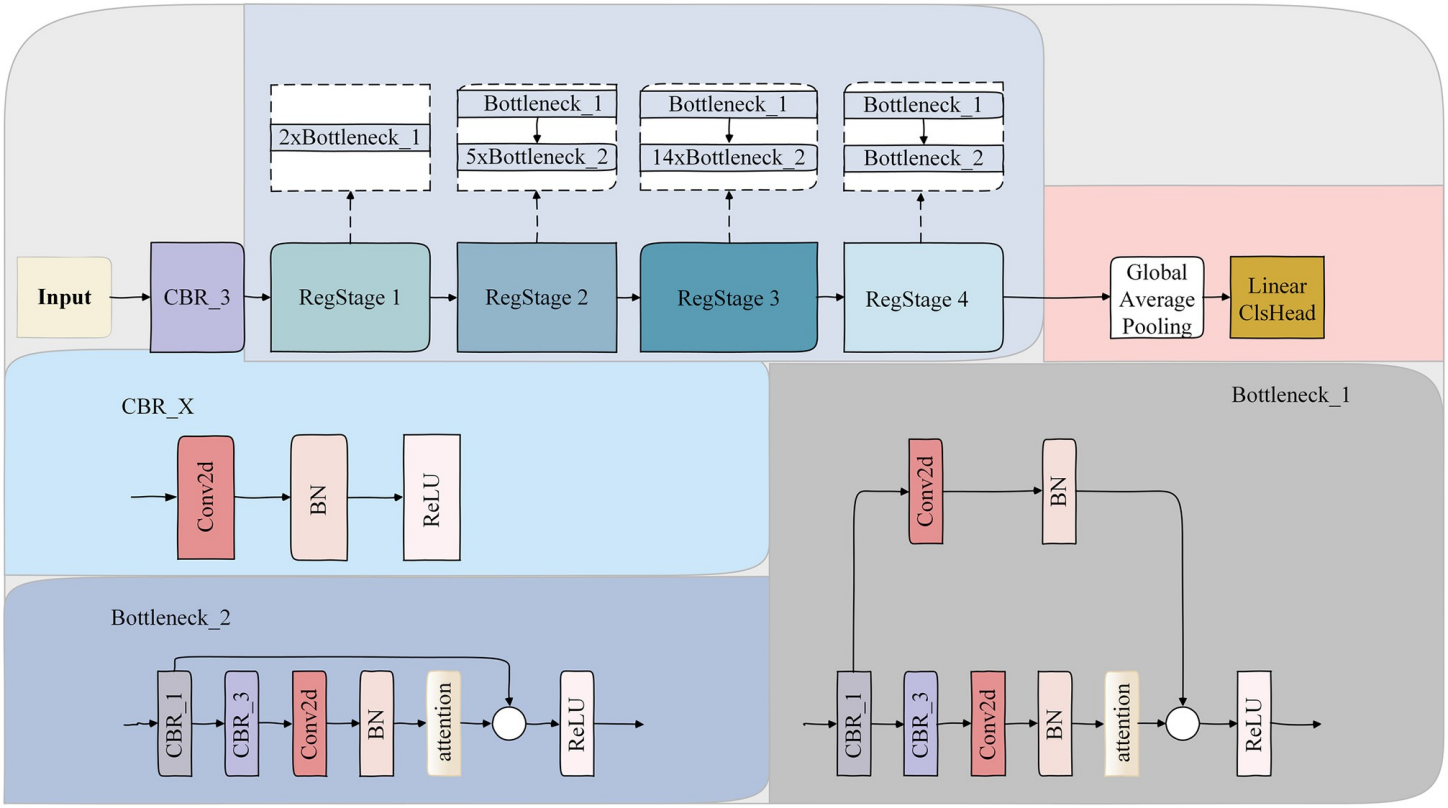
Architecture of the RegnetX-3.2gf network and integration locations of the attention mechanisms. CBR_x: kernel size, with 1 denoting 1 × 1, and 3 denoting 3 × 3. The contents of the dashed box represent the composition of each stage.

The visual attention mechanism is a brain signal processing mechanism unique to human vision, and the attention mechanism in deep learning is based on the human visual attention mode of thinking. When humans observe their surroundings, they rapidly scan the entire panorama before focusing on specific areas of interest, guided by neural signal processing. This process culminates in the formation of an attention focus, enabling the extraction of the most pertinent information. To investigate the impact of attention mechanisms on cell classification performance, we implemented and evaluated various types of attention mechanisms within cell classification tasks. Detailed parameters of these attention mechanisms are shown in Table 3, and the integration locations are illustrated in Fig 3. Each attention mechanism was individually incorporated into the proposed model (RegnetX-3.2gf).

**Table 3:** Attention mechanisms.

Attention mechanism	Abbreviation	Type
Squeeze-and-Excitation [35]	SE	channel attention
Efficient Channel Attention [36]	ECA	channel attention
Efficient Multi-Scale Attention [37]	EMA	multiscale attention
Convolutional Block Attention Module [38]	CBAM	mixed attention
Normalization-based attention module [39]	NAM	mixed attention
Coordinate Attention [40]	CA	spatial attention
Simple Attention Module [41]	SimAM	self-attention

### Category activation mapping

Category activation mapping (CAM), also known as category heat maps, is an essential tool for visualizing the focal points of the model training process. It enhances the understanding and analysis of neural network functionality and aids in evaluating the accuracy of decision-making. By using gradient information from the convolutional layer, heat maps are generated to identify areas of interest for each neuron, emphasizing the focal regions of the image. The color gradients in the category activation map represent the learning focus of the model, with darker shades indicating higher weights and red-highlighted regions denoting the primary focus of the model.

When experts on morphological features classify cells, they consider multiple factors such as the nucleus, cytoplasm, and their shape, size, and color, among others. However, the nucleus is one of the key features to distinguish cell types [42]. This study used Grad-CAM, proposed by Selvaraju et al. [43], to generate category activation maps and determine where the model’s focus on. Comparing whether the regions highlighted during model categorization correspond approximately with those identified by experts on morphological features and determining whether the model is reliable when making classification decisions.

## Results

### Implementation details and evaluation indicators

All the analyses were conducted using the Windows operating system, and the neural network model was trained using the PyTorch software library and NVIDIA GeForce RTX 3090. The criteria used for performance evaluation were Top-1 and Top-5 Accuracy, Precision, Recall, and F1 Score. The mathematical formulations for these metrics are provided in Eqs 7 to 10.


$$Accuracy=\frac{TP+TN}{TP+TN+FP+FN}$$ (7)

$$Precision=\frac{TP}{TP+FP}$$ (8)

$$Recall=\frac{TP}{TP+FN}$$ (9)

$$F1=\frac{2\times Precision\times Recall}{Precision+Recall}$$ (10)

In this context, TP, TN, FP, and FN denote true positives, true negatives, false positives, and false negatives, respectively. TP and TN represent the numbers of category images that are correctly classified and correctly unclassified based on the actual scenario, respectively, whereas FP and FN indicate the numbers of category images that are incorrectly classified and incorrectly unclassified, respectively.

From the above aforementioned formula, it is evident that the accuracy rate reflects the percentage of correct predictions, the precision rate indicates the percentage of accurately predicted positive identifications, and the recall rate represents the percentage of actual positive identifications that were correctly predicted. Both accuracy and recall are essential metrics for evaluating model performance; however, they often exhibit an inverse relationship. Therefore, a reconciliatory metric is necessary to jointly consider these factors, for which F1 Score is used. Particularly in the context of addressing category imbalance, F1 Score enables a more comprehensive and equitable assessment of model performance, serving as a critical guiding principle for model selection.

### Comparison of the results

#### Comparative analysis of the network models

Table 4 presents the average values of accuracy, precision, recall, and F1 Score for the BM dataset on the five common classification models, highlighting the best results. This study first compared the impact of utilizing pre-trained weights against not using them on classification outcomes. As shown in Table 4, employing pre-trained weights from the ImageNet models enhanced classification performance for each model. Notably, Swin Transformer V2 yielded an F1 Score of 67.499% when pre-trained weights were used, demonstrating the highest performance among the models. However, this model has a significantly high number of parameters, necessitating a more robust training environment and longer training duration than the other models. In contrast, the lightweight Regnet model yielded an F1 Score that was only 3.274% lower than that of Swin Transformer V2, and its number of parameters was less than one-fifth of that of Swin Transformer V2. Regnet is superior to other models in computational speed, portability, and equipment requirements, making it more suitable for practical applications. Therefore, this study recommends using Regnet and performs the subsequent analyses by using this model.

**Table 4:** Results of the model comparison.

Model & pre-training		Top1-accuracy	Top5-accuracy	Mean Precision	Mean Recall	Mean F1 Score
**Densenet-201**	Random	82.491	98.340	65.425	61.980	62.728
	ImageNet	83.048	98.267	67.677	64.450	65.695
**Resnext50**	Random	82.271	98.283	66.114	60.618	62.151
	ImageNet	82.814	98.114	68.393	62.898	65.041
**MobileNetV3**	Random	80.350	97.873	59.065	52.881	54.950
	ImageNet	83.052	98.379	70.373	59.594	62.502
**Regnet**	Random	82.173	98.200	65.461	59.749	61.374
	ImageNet	82.717	98.240	68.355	61.989	64.225
**Swin Transformer V2**	Random	82.521	98.429	63.308	60.609	63.129
	ImageNet	**84.214**	**98.608**	**73.221**	**64.644**	**67.499**

The results are presented as percentages. The training from scratch is referred to as "random" and the use of pre-trained weights is indicated as "ImageNet."

#### Comparison of the loss functions

**Fig 4:**
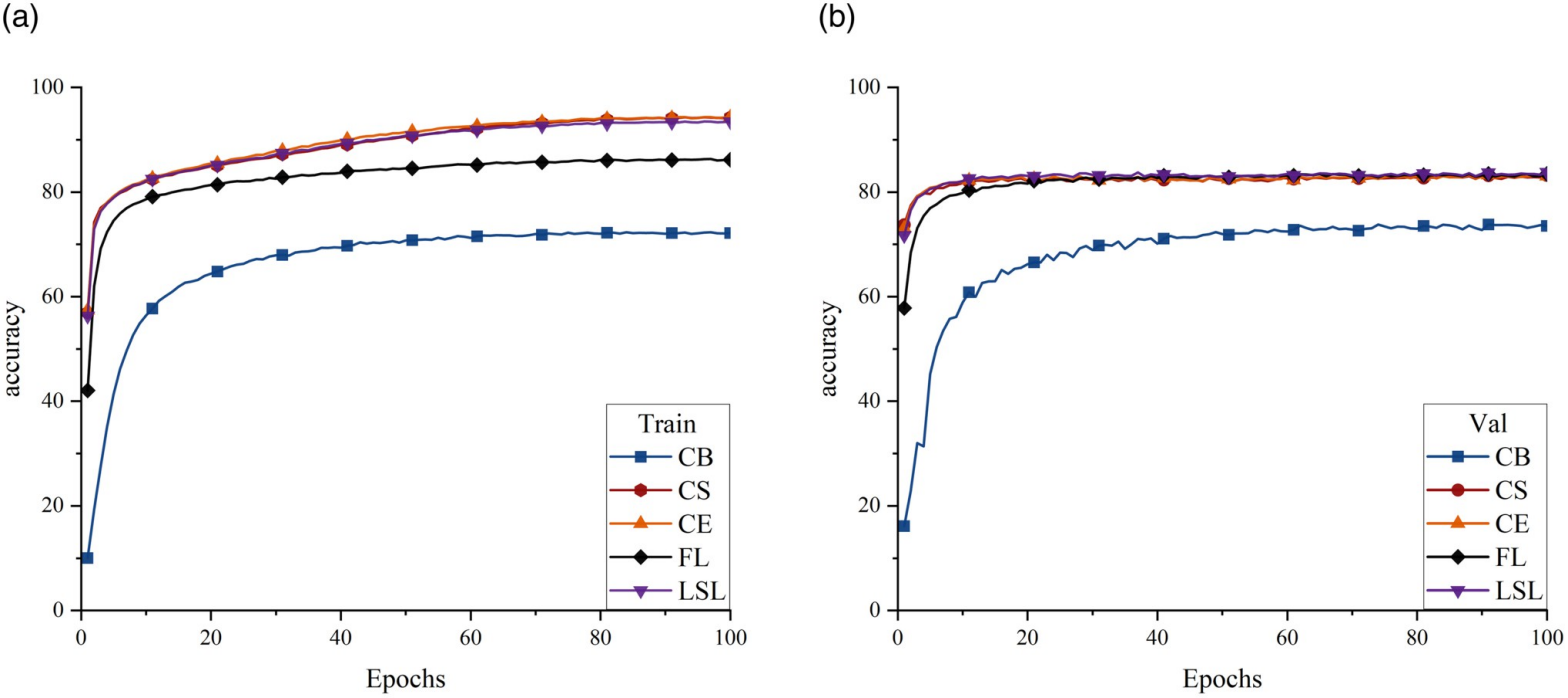
Accuracy curves for the five loss functions, represented by different colors and shapes of the folds. (a) Training-set accuracy. (b) Validation-set accuracy.

The loss functions employed in this study to mitigate category imbalance can be categorized into the following two primary groups: those based on the CE loss function and those based on the FL function. As illustrated in Fig 4, both the FL and the CB loss derived from the FL exhibited a tendency to plateau at relatively low values. Additionally, both losses overall demonstrated poor classification performance, with the CB loss showing the worst performance, yielding a F1 Score of only 49.305% (Table 5). This phenomenon can be attributed to an excessive focus on a limited subset of samples, thereby neglecting others and ultimately deteriorating overall classification performance. Conversely, CE loss and its derivatives LSL and CS loss showed better performance. Although the CE loss function exhibited significant overfitting on the validation set, the implementation of the LSL function resulted in a more gradual reduction in training phase loss, mitigating network overfitting to some extent and achieving a precision rate of 71.719%. Moreover, the CS loss function, stemming from the CE loss function, reduced the overfitting by incorporating a penalty mechanism, yielding the highest F1 Score (65.145%). Furthermore, accuracy and recall also improved to 67.323% and 64.083%, respectively. Consequently, this study used the CS loss function in the subsequent analyses.

**Table 5:** Results of the comparison of the loss functions.

	Top1-accuracy	Top5-accuracy	Mean Precision	Mean Recall	Mean F1 Score
**CE**	82.717	**98.240**	68.355	61.989	64.225
**LSL**	**83.163**	97.518	**71.719**	62.064	64.460
**CS**	82.626	97.733	67.323	64.083	**65.145**
**FL**	82.928	98.179	63.396	57.931	60.078
**CB**	73.288	95.649	47.686	**65.736**	49.305

The results are presented in percentages. CE: cross-entropy loss. LSL: label-smoothing loss. CS: cost-sensitive loss. FL: focal loss. CB: category-balance loss

#### Comparison of the attention mechanisms

The utilization of attention mechanisms is a widely employed strategy to enhance model performance and refine classification accuracy. However, their effectiveness may not be uniform across all tasks. The integration of attention mechanisms, except for channel attention SE, frequently reduced the classification performance instead of enhancing it (Table 6). This phenomenon occurs when an image contains a substantial amount of irrelevant information, as the presence of attention mechanisms may lead to the acquisition of incorrect information, thereby diminishing overall performance.

**Table 6:** Results of the comparison of the attention mechanisms.

		Top1-accuracy	Top5-accuracy	Mean Precision	Mean Recall	Mean F1 Score
**Cost sensitive Loss**	Base	82.626	97.733	67.323	64.083	65.145
	SE	82.929	97.870	**68.183**	63.722	**65.155**
	ECA	**83.119**	**97.918**	67.960	62.934	64.785
	EMA	82.468	97.767	66.962	63.402	64.708
	CBAM	82.999	97.866	67.129	62.978	64.572
	CA	82.926	97.729	67.100	61.931	63.616
	SimAM	82.651	97.731	68.167	61.102	63.397
	NAM	77.796	90.985	47.803	45.744	46.197

The results are presented as percentages.

**Fig 5:**
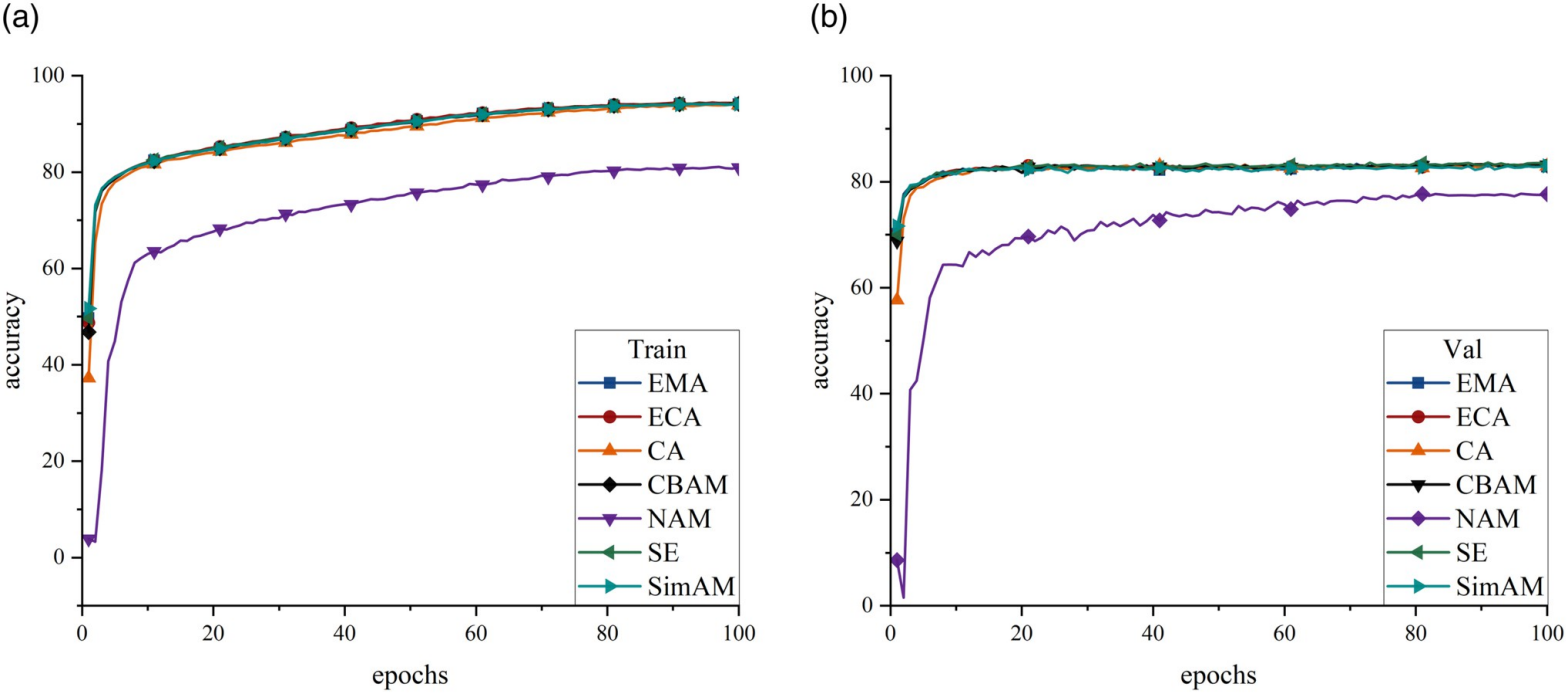
Accuracy curves of the seven attention mechanisms, represented by different colors and shapes of the folds. (a) Training-set accuracy. (b) Validation-set accuracy.

The channel attention mechanism evaluates the significance of each channel within the feature map and assigns appropriate weights, enabling the network to concentrate more effectively on pertinent feature channels and reducing the influence of noise. Notably, the SE mechanism demonstrated the most rapid rate of increase, yielding an F1 Score of 65.154%, thereby surpassing the performance of the baseline model (Fig 5).

**Fig 6:**
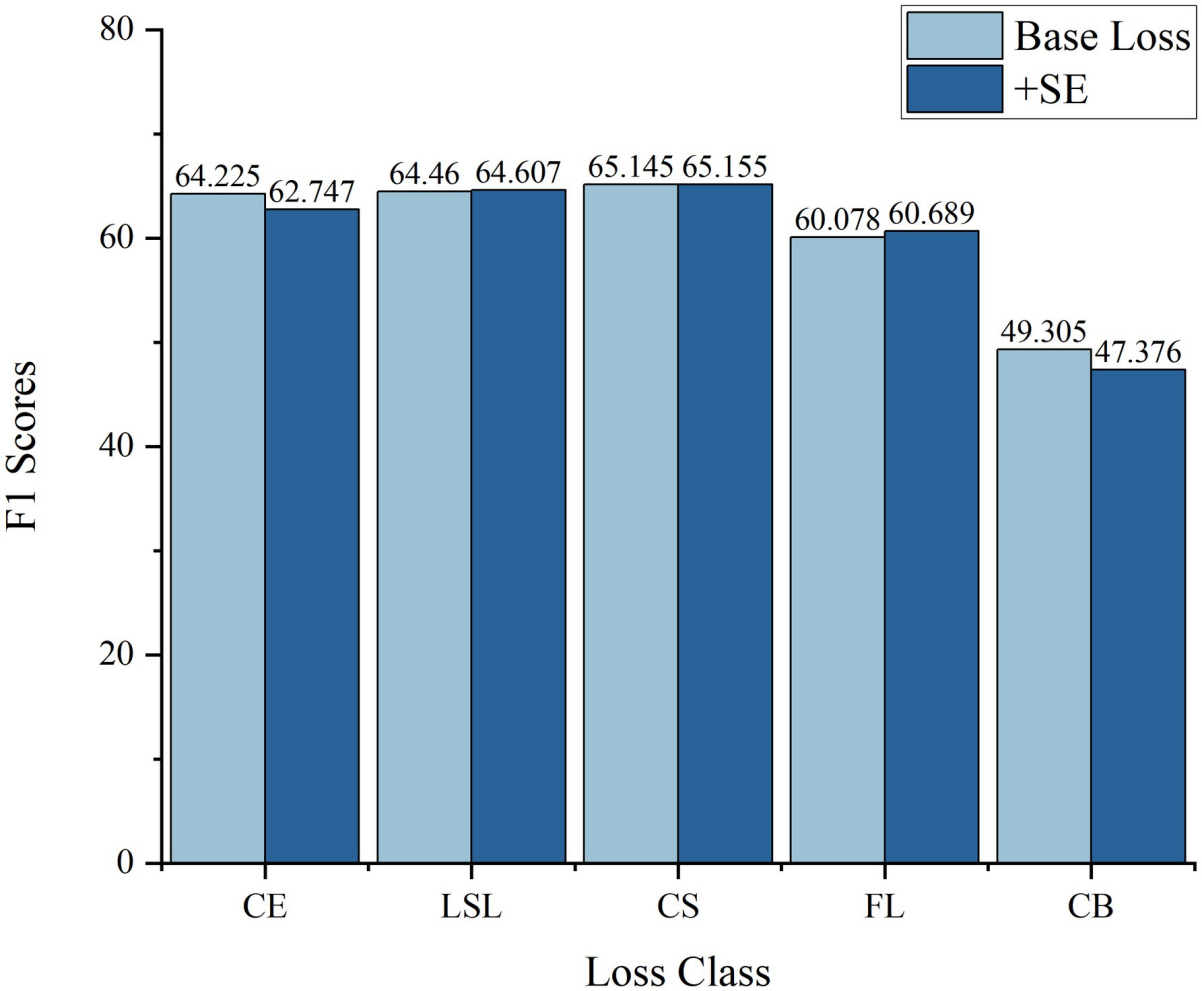
Comparison of the F1 Scores before and after incorporating the attention mechanism for different loss functions. The results are shown as percentages.

This study evaluated the SE attention mechanism across five distinct loss functions (Table 7 and Fig 6). The findings indicate that the incorporation of SE led to only marginal improvements in the classification performance of the model. Additionally, performance declines were observed in the CE and CB metrics. Therefore, before using attention, the image quality should be improved, the additional information should be removed, and its influence on the attention mechanism should be reduced.

**Table 7:** Results of incorporating the SE attention mechanisms for various loss functions.

Loss+SE	Top1-accuracy	Top5-accuracy	Mean Precision	Mean Recall	Mean F1 Score
LSL	**83.409**	97.630	**70.650**	62.510	64.607
CB	71.452	95.261	46.393	62.940	47.376
FL	83.200	98.277	65.483	58.303	60.689
CS	82.929	97.870	68.183	**63.722**	**65.155**
CE	82.903	**98.386**	68.380	60.22	62.747

The results are presented as percentages. SE: Squeeze-and-Excitation.

#### Results and discussion

To demonstrate that the cost-sensitive loss function and SE attention module are effective not only in the RegNet model but also in other classification models, these methods were applied to four additional models. The SE module was added in a similar position to RegNet. Notably, since MobileNetV3 already includes the SE module, we only tested the effect of the cost-sensitive loss function.

**Fig 7:**
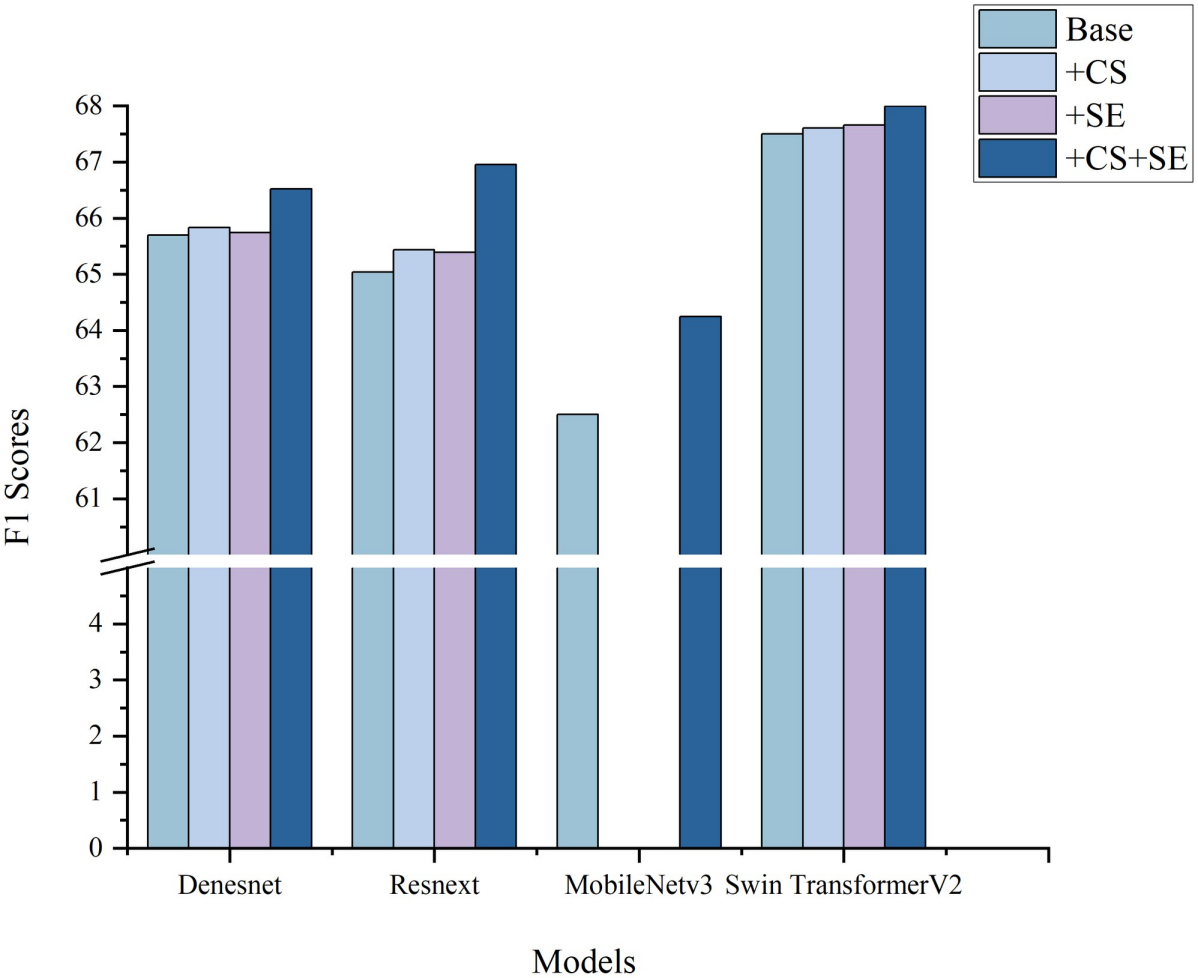
Comparison of the F1 Scores before and after incorporating CS and SE individually or together. The results are shown as percentages. CS: Cost-Sensitive. SE: Squeeze-and-Excitation.

The use of the cost-sensitive loss function and SE attention module resulted in varying degrees of improvement in classification performance across all the models, with ResNeXt50 showing the highest increase in performance (Fig 7). Table 8 displays a comparison of the improved models alongside the original dataset, providing classification results. The improved RegNet outperformed the original dataset classification result, with an F1 Score increase of 10.655%. The other models also exhibited varying degrees of improvement, suggesting that the cost-sensitive loss function and SE attention module have strong applicability and effectiveness across various models.

**Table 8:** Results of incorporating CS and SE for various models, compared with the original dataset provided results.

Mean	precision	recall	F1 score
Densenet-201	68.292	65.831	66.524
Resnext50	70.268	65.140	66.953
MobileNetV3	70.980	61.328	64.249
Swin Transformer V2	**73.370**	65.020	**67.992**
Regnet	68.183	63.722	65.155
Matek	51	**68.9**	54.5

The results are presented as percentages. CS: Cost-Sensitive. SE: Squeeze-and-Excitation.

**Fig 8:**
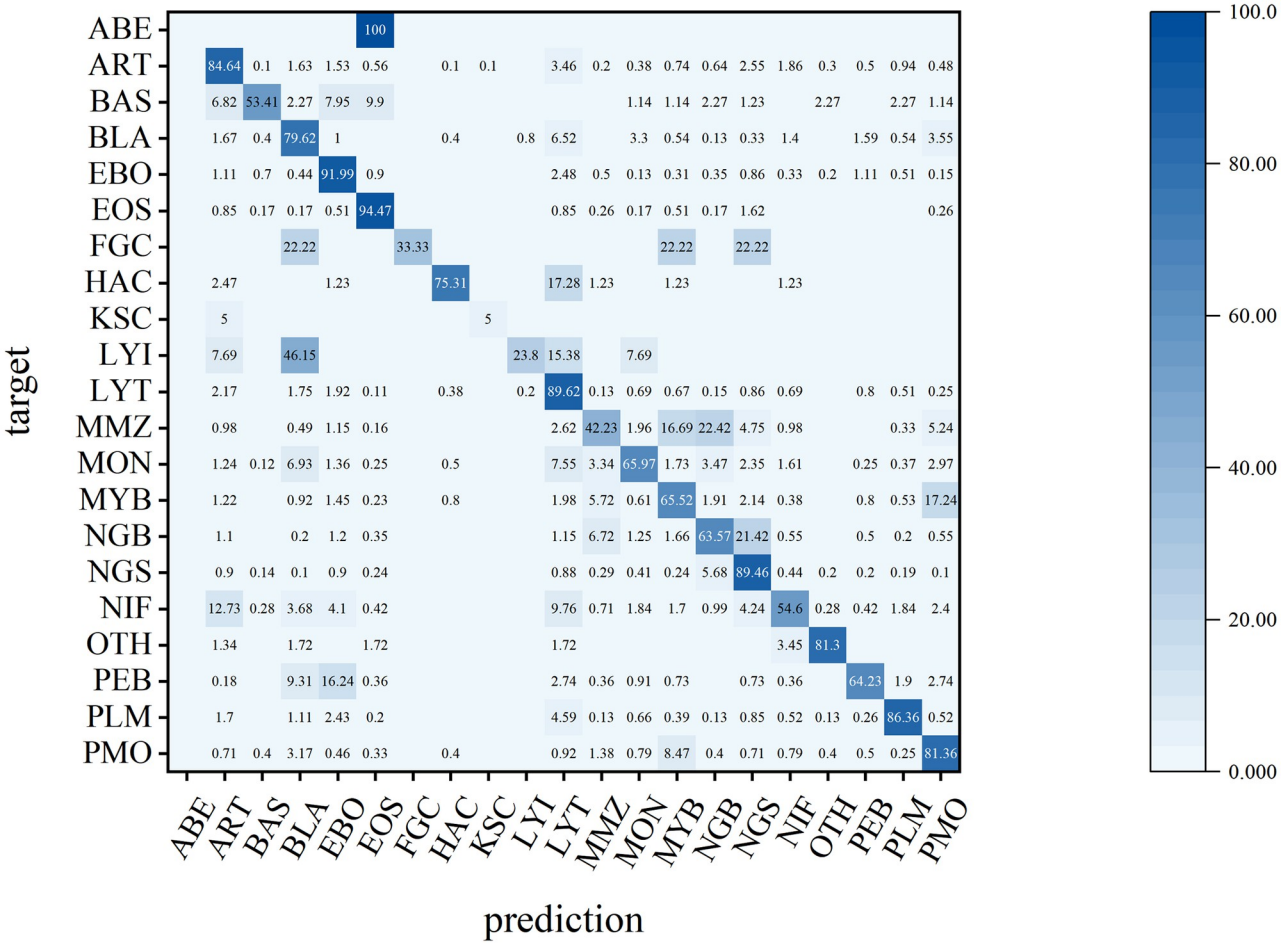
Confusion matrix.

To gain a deeper understanding of the cell types that are difficult for the training model to distinguish, the classification results were visualized using confusion matrix analysis (Fig 8). Categories such as ABE, BAS, FGC, KSC, LYI, MMZ, and NIF exhibited relatively poor classification performance. This observation can be attributed to several factors.

Primarily, these categories have small sample sizes, causing the model to be biased toward categories with large sample sizes during the training phase. Consequently, the performance and classification accuracy for the categories with small sample sizes are reduced. For example, the dataset analyzed in this study included only eight images of abnormal eosinophils. Secondly, it is challenging to accurately classify similar cell types, such as abnormal eosinophils being misclassified as eosinophils, where the morphological differences are minimal and the number of abnormal eosinophils is very low. Furthermore, the continuous nature of cell growth and development leads to a lack of distinct boundaries among cell types, complicating the classification process. Cells with low original differentiation and high similarity are prone to misclassification, such as lymphoblasts misidentified as myeloblasts, and cells from adjacent developmental stages with high similarity may be incorrectly categorized, such as promyelocytes misidentified as myelocytes.

To investigate how the cost-sensitive loss function and SE attention mechanism enhance model classification performance, we compiled F1 Scores by category (Table 9). Implementing the CS loss function resulted in varying levels of improvement in F1 Scores across nearly all the categories. In particular, the four low-sample categories—FGC, KSC, LYI, and OTH—showed the most notable improvements, which showed poor performance in the confusion matrix analysis. The principle of the cost-sensitive loss function was to use a cost matrix to quantify the cost of misclassification in different categories, which made the model pay more attention to those categories with higher misclassification costs. The model learned fewer features during training because of the small number of samples in these small sample categories, resulting in a higher rate of classification error, and the cost-sensitive loss function is just designed to address this, the classification accuracy of the model is improved by increasing the accuracy of the few sample categories without reducing the accuracy of other categories.

**Table 9:** Results for each category when CS and SE are applied individually or together.

Class	Base	Base+SE	Base+CS
NGB	65.623	65.734	65.302
NGS	87.67	88.008	87.767
LYT	87.508	86.712	87.285
MON	66.997	68.803	67.25
EOS	93.427	93.43	93.514
BAS	57.51	58.05	58.873
MMZ	40.468	38.536	40.543
MYB	65.069	66.903	63.748
PMO	79.728	80.096	79.911
BLA	80.334	79.261	80.752
PLM	87.753	86.506	87.903
KSC	36.362	32.054	45.977
OTH	74.481	74.154	78.418
ART	85.317	85.872	85.579
NIF	55.968	57.418	56.704
PEB	70.287	72.09	71.19
EBO	91.3	91.375	91.304
HAC	63.742	62.695	61.859
ABE	0	0	0
LYI	17.081	0	19.538
FGC	42.098	29.954	44.604

The results are presented as percentages.

As shown in Table 9, the effects of the SE attention mechanism were also consistent across all the categories. The SE attention mechanism functions by capturing dependencies between different channels and assigning appropriate weights to each, thereby enhancing feature representation and boosting model performance. In the BM dataset, where categories exhibit high similarity and share many channels, the integration of the SE attention mechanism results in uniform improvement across all the categories.

### Activation graph visualization and analysis

**Fig 9:**
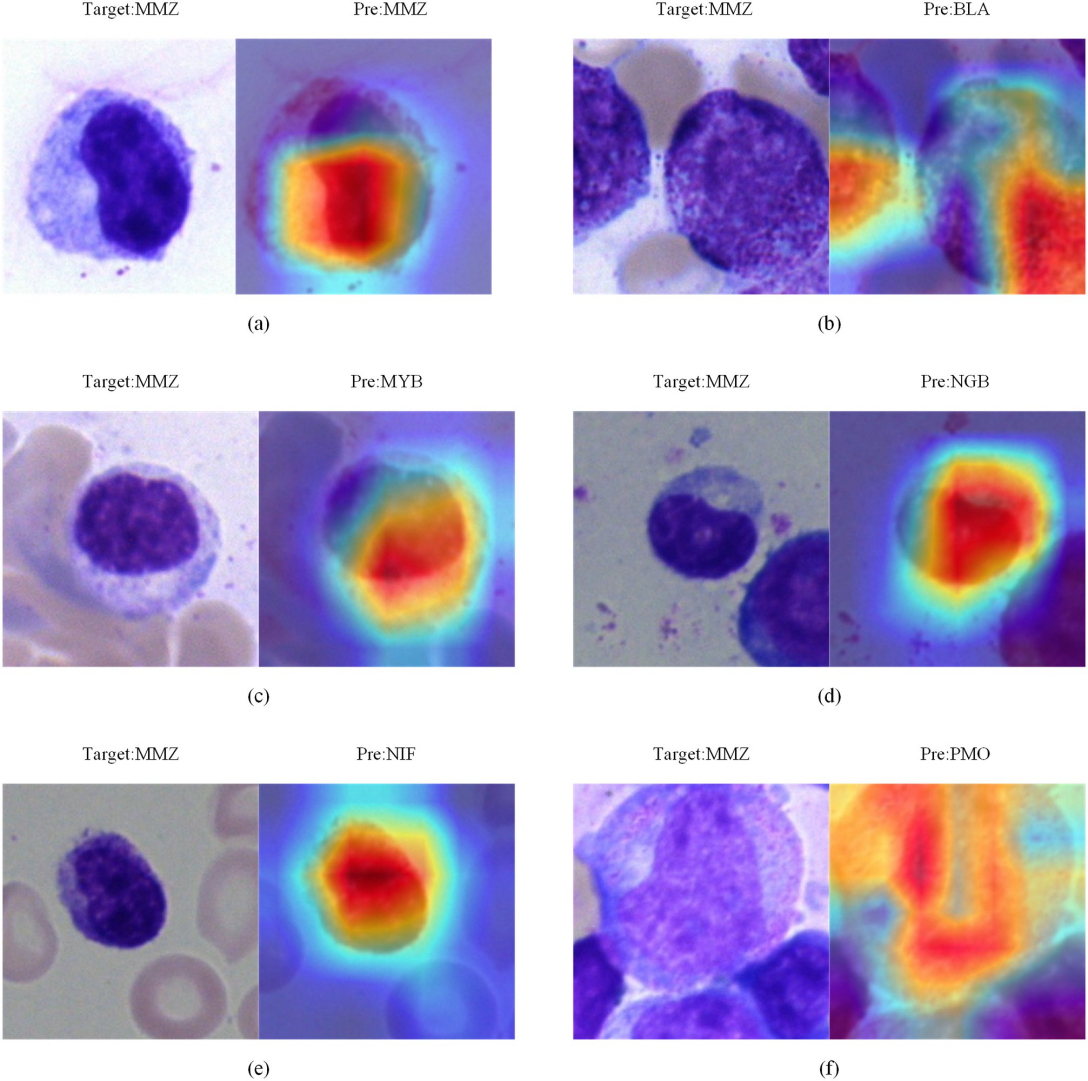
MMZ activation maps. (a) is correctly categorized, (b)(c)(d)(e)(f) are misclassified.

**Fig 10:**
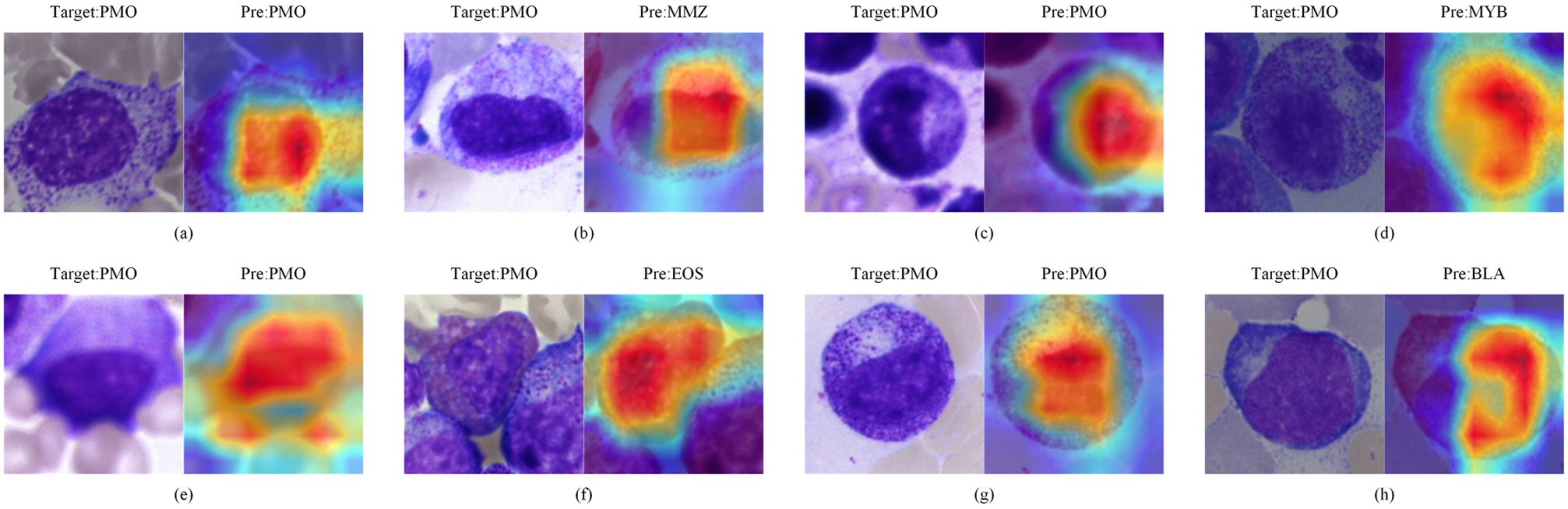
PMO activation diagram. (a)(c)(e)(g) are correctly categorized, (b)(d)(f)(h) are misclassified.

The Grad-CAM analysis conducted in this study focused on the following two crucial cell types in AML classification: Promyelocytes (PMO) and Metamyelocyte (MMZ). The illustrations of accurate and inaccurate categorizations, along with their corresponding category activation mapping diagrams, are presented in Figs 9a–9f and 10a–10h.

MMZ cannot undergo cell division and are smaller than myelocytes. They exhibit a reduced nuclear size, occupying less than half of the whole cell, and display a one-sided concavity or kidney-shaped morphology, although the concavity is less pronounced than that of the nuclear neck (Fig 9). In contrast, promyelocytes are larger than their precursor cells, with relatively smaller nuclei that occupy more than half of the whole cell. The nuclei of promyelocytes are round or ovoid and are slightly eccentrically located (Fig 10). The activation diagram indicated that the correctly identified cells maintained consistent morphological characteristics, accurately reflecting the rough shapes of both cells and nuclei (Figs 9a and 10a). Conversely, the misclassification instances revealed that the presence of contaminating cells within the image can interfere with the target cell (Figs 9b and 10f). This interference causes the model to concentrate on incorrect features, ultimately resulting in misclassification. For example, misclassifications illustrated in Figs 9c and 10b arose from the model confusing chromatin with the nucleus, leading to a shift in focus and subsequent erroneous categorization of cells.

The activation mapping diagrams showed that the model focused on the cells themselves when making classifications, especially on the nucleus and its shape, rather than the surrounding interfering cells. This feature was approximately the same as the features assessed by doctors. Thus, the model was reliable when making classification.

## Conclusion

This study was performed with the ultimate goal of advancing the auxiliary diagnosis of AML. In response to the issue of imbalanced bone marrow cell types, we evaluated the performance of common classification models, loss functions, and attention mechanisms on BM datasets. We analyzed the impacts of these factors on cell classification tasks, combined with visual analysis and practical applications, ultimately proposing a solution suitable for addressing the imbalance in bone marrow cell classification, serving as a reference for future research. The optimal model (RegNetX-3.2gf + CS + SE) yielded average precision, recall, and F1 Score of 68.183%, 63.722%, and 65.155%, respectively. This model outperformed the original dataset results by 17.183% in precision and 10.655% in F1 Score.

This comprehensive and systematic evaluation enhances the generalizability and applicability of research results and may serve as a reference for future studies and practical applications, potentially facilitating the application of models in clinical settings. However, the study has limitations, as the dataset used is relatively homogeneous, featuring identical cell processing methods. Tests with a more diverse set of data sources should be performed in the future. Additionally, future models should focus on distinguishing more details, such as monocytes, promonocytes, and monoblasts, to be more effective in medical practice. There is considerable room for improvement in the classification accuracy of models. Accordingly, we plan to increase the dataset size through methods such as data augmentation, expansion, and data collection from multiple hospitals, aiming to enhance the quality of images. For AML subtype task, irrelevant cell types will be filtered out to further refine the model and improve classification accuracy. By collaborating with domain experts and integrating practical applications, we will seek guidance on the key challenges in bone marrow cell classification, enabling targeted improvements in future research endeavors.
